# DFA7, a New Method to Distinguish between Intron-Containing and Intronless Genes

**DOI:** 10.1371/journal.pone.0101363

**Published:** 2014-07-18

**Authors:** Chenglong Yu, Mo Deng, Lu Zheng, Rong Lucy He, Jie Yang, Stephen S.-T. Yau

**Affiliations:** 1 Mind-Brain Theme, South Australian Health and Medical Research Institute, Adelaide, South Australia, Australia; 2 Quant Investment Department, Huashang Fund Management Co., Ltd., Beijing, China; 3 Electrical and Computer Engineering and CyLab Mobility Research Center, Carnegie Mellon University, Moffett Field, California, United States of America; 4 Department of Biological Sciences, Chicago State University, Chicago, Illinois, United States of America; 5 Department of Mathematics, Statistics, and Computer Science, University of Illinois at Chicago, Chicago, Illinois,United States of America; 6 Department of Mathematical Sciences, Tsinghua University, Beijing, China; University of Memphis, United States of America

## Abstract

Intron-containing and intronless genes have different biological properties and statistical characteristics. Here we propose a new computational method to distinguish between intron-containing and intronless gene sequences. Seven feature parameters 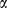, 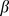, 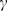, 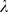, 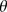, 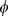, and 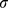 based on detrended fluctuation analysis (DFA) are fully used, and thus we can compute a 7-dimensional feature vector for any given gene sequence to be discriminated. Furthermore, support vector machine (SVM) classifier with Gaussian radial basis kernel function is performed on this feature space to classify the genes into intron-containing and intronless. We investigate the performance of the proposed method in comparison with other state-of-the-art algorithms on biological datasets. The experimental results show that our new method significantly improves the accuracy over those existing techniques.

## Introduction

An important problem for geneticists as well as computer scientists involves classifying particular items into common groups. Here we focus on classifying gene sequences as either intron-containing or intronless. Intron-containing and intronless genes have different biological properties and statistical characteristics. For example, congruent with the Spearmans rank correlation, the comparison of intron-containing and intronless genes shows significantly reduced expression for intronless genes when compared to intron-containing genes [1]. Furthermore, intron-containing and intronless genes usually play important roles in evolution of proteins [2]–[4]. These observations raise interesting questions about the classification of intron-containing and intronless genes.

Peng et al. [5] have discovered that long-range correlation exists in the intron-containing genes but does not exist in the intronless genes. This work was based on a simple random-walk model of gene sequences, in which a pyrimidine led to a step up and a purine a step down. Consequently, the walk resulted in a definite landscape for a given sequence and only one parameter was calculated based on the landscape. This parameter was proposed to distinguish between the intron-containing and intronless genes. However, further study showed that this finding can not be used as a general method to identify intronless genes [6], [7]. Zhang et al. [7], [8] introduced a Z-curve consisting of three parameters. As an application, they used the Z-curve method to classify a dataset consisting of 100 intron-containing and 100 intronless genes. The discriminant accuracy as high as 89.0% can be obtained by using Fisher's linear discriminant algorithm based on Z-curve. However, although the distributions of three different biological types were displayed in Z-curve, it did not reveal the cross-correlations of distances between the nucleic bases, which are also important parameters to classify genes into intron-containing and intronless. In a similar way, Ma [9] created a model based on position weight function to describe genes by transforming them into quaternary numbers. Especially, this method indicates that E.coli K12s genome and the eukaryote yeasts genome have different strengths of single nucleotide periodicities. Yau et al. [10] firstly developed two-dimensional DNA graphical representation without degeneracy. Since then Yau and his collaborators have been studying efficient methods to cluster and classify DNA and proteins [11]–[18].

Some successful programs for exon/intron parsing are also proposed. For example, GENSCAN [19] was shown to be dramatically more accurate than the previous state-of-the-art prediction algorithms. It is based on a generalized hidden Markov model (GHMM) framework, and remains a popular bioinformatics tool. More recent de novo gene predictors have also been created, including N-SCAN [20] and EXONSCAN [21]. De novo gene predictors additionally made use of aligned gene sequence from other genomes [22]. Alignments can increase predictive accuracy since protein-coding genes exhibit distinctive patterns of conservation. These modern gene-finding or gene-parsing systems provide a prediction of precise (predicted) splice sites of the exons/introns in genes, while also producing the intron-bearing status of genes.

Here we propose a new approach, DFA7, to classify genes as to their intron-bearing status. We investigate three new parameters which are based on the cross-correlations between the distributions of distances of nucleic bases in gene sequences. Those new parameters together with Zhang et al. 's original three parameters [7] and the value of their total standard deviation can be used to significantly improve the accuracy of classification on intron-bearing status of genes. We perform our DFA7 method on three large gene datasets. The experimental results show that our method significantly improves the discriminant accuracy over those existing techniques. In addition, we examine our 7-dimensional feature vector by one-by-one feature deletion, and compare the SVM's efficiency with other machine learning approaches.

## Materials and Methods

### Background

The Z-curve theory of DNA sequences was firstly developed by Zhang et al. [7], [8]. Consider a DNA sequence with 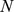 bases. Let the number of steps be denoted by 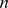 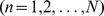. We count the cumulative numbers of base A, C, G, T which occur in the subsequence from the first to the 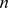
th base in the DNA sequence. The cumulative numbers are denoted by 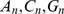 and 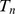, respectively. The Z-curve is a three-dimensional curve which consists of a series of nodes 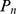 (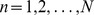), whose coordinates are denoted by 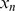, 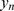 and 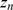. It is shown that
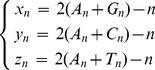
where 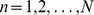 and 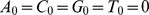. The connection of the nodes 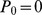, 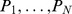 one by one by straight lines is defined as the Z curve of the DNA sequence.

Detrended fluctuation analysis (DFA), firstly introduced by Peng et al. [5], is a scaling analysis method used to estimate long-range power law correlation parameters in noisy signals. By using this technique, Zhang et al. [7] calculated three exponents 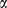, 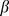, and 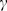 for a given sequence based on its Z-curve. A 3-dimensional space is spanned by the three exponents. Each DNA sequence may be represented by a point in this space. For any query gene sequence, calculate its 3 exponents 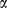, 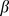, and 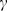, corresponding to a point in the 3-dimensional space. If the point is situated at the upper region of the separating plane, the gene is discriminated as an intronless one; otherwise, the gene is an intron-containing one.

For pursuing higher classification accuracy, more intrinsic parameters are needed. Here we propose a novel method, DFA7 method. In this approach, we introduce 4 new feature parameters 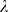, 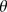, 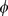, and 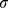 for each DNA sequence based on DFA. Combining with 3 known parameters 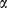, 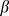, and 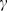 from Z-curve we can generate a 7-dimensional feature space, which can be used to classify gene sequences into intron-containing and intronless with much higher discriminant accuracy.

### DFA7 method

In a DNA sequence, 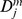 represents the cumulative distance of all nucleotides of nucleic base 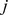 (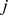 = 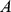, 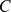, 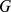, 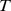) to the first nucleotide (regarded as origin) in 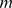 steps. Let 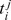 be the distance from the first nucleotide to the 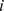
th nucleotide if the 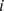
th nucleotide is 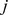, otherwise; 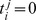. Thus 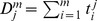. For example, 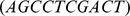 is a DNA sequence. For nucleic base 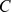, 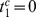, 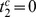, 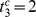, 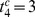, 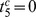, 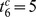, 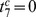, 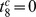, 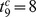, 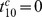, so 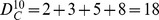. Similarly, we get 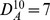, 
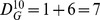
 and 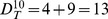. Thus three types of cumulative distances can be defined as follows:
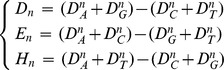
where 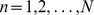 and 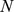 is the length of the DNA sequence. 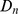, 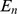, and 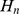 reveal the cross-correlation of the “position” of each nucleic base in a DNA sequence. These cumulative distances are natural objects from the original DNA sequence, and embody more sequence information which Z-curve fails to provide.

Now we construct a 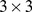 cumulative distance matrix based on 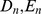 and 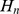, and then use this matrix to compute 3 new feature parameters 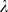, 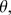 and 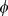. The algorithmic steps of setting the new parameters 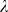, 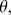 and 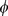 are provided as follows:

1. Set a window with width 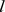, 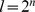, 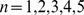, and move the window from the site 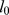.
2. Calculate the variation of each distribution at the two ends of the window,
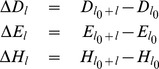

3. Shift the window sequentially from the beginning site 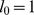 to 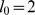 and so on, up to 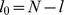, where 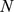 is the length of the sequence. For each value of 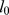 starting from 1 to 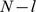, calculate each corresponding 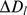, 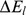, 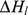.
4. Define the fluctuation functions
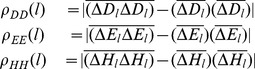


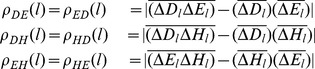
where the bars indicate an average over all site 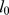 in the sequence. Then the matrix of fluctuation functions is defined as follows:
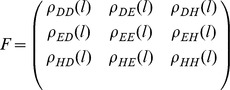
Obviously, 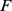 is a real and symmetric matrix. Denote the three eigenvalues of 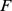 by 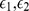 and 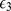, such that 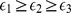. Based on fluctuation analysis, we can get that
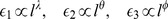
where 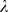, 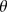, and 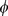 are three parameters determined by the slopes in the log-log plots. In other words, 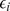 (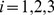) is a proportional function of 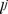 (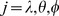), i.e., 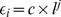, 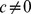. Because of the nonlinear scaling of the axes, a function of the form 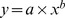 will appear as a straight line on a log-log graph, in which 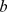 is the slope of the line. Therefore, the parameters 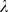, 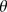, and 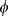 can be computed by estimating each slope of log-log graph corresponding to 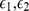 and 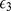 from the numerical data.
5. Estimate the slopes 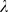, 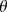, and 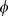 of each log-log graph corresponding to 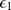, 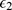, and 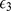 computed in step (4).

Thus, for any given DNA sequence, we can calculate three parameters 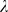, 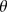, and 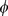 by using the above five algorithmic steps. In step (1), in order to reduce the error for determining the slope 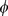 and improve the computational efficiency, the values of 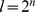 (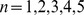) are adopted. The line fitted by those 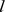
's is perfect. Even if the linearity is not so perfect in several cases, the squared error with respect to the slope and intercept parameters is minimized and the unique straight line can also be obtained by performing a least-squares fit of the data.

After determining 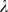, 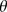, and 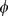, we have a 7-dimensional feature vector consisting of parameters 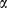, 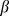, 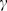, 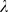, 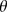, 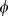, and 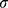, where 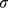 is the sample standard deviation of the first 6 features. Then a machine learning method based on a support vector machine (SVM) equipped with a Gaussian radial basis kernel function (RBF) is used for prediction of intronless and intron-containing genes based only on the primary sequences.

**Figure 1:**
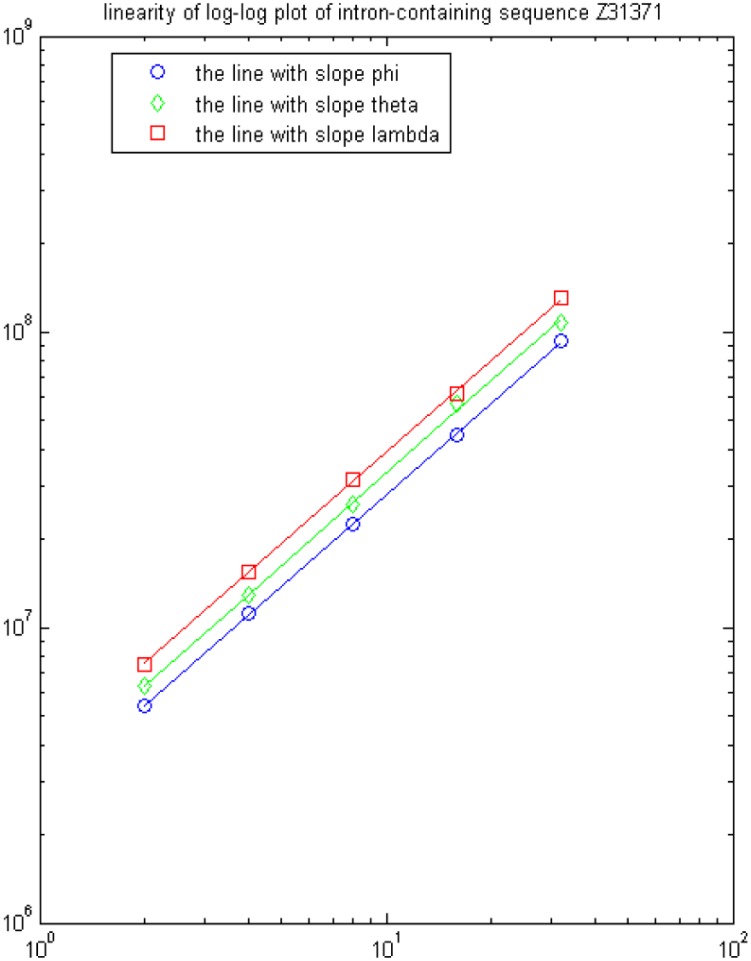
Linearity of log-log plots of three feature parameters 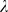, 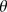, and 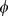 based on gene Z31371.

**Figure 2:**
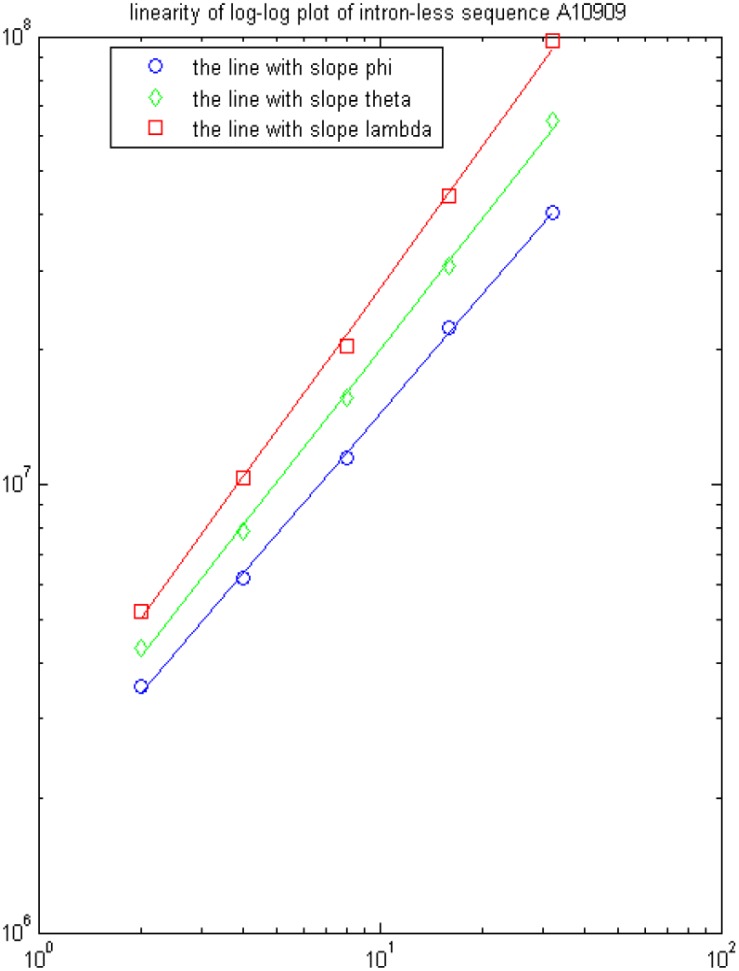
Linearity of log-log plots of three feature parameters 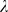, 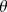, and 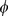 based on gene A10909.

We pick up 12 gene sequences as an illustration. The corresponding 7 feature parameters are calculated and listed in Table 1. The first 6 genes are intronless and the last 6 are intron-containing. Then an optimal hyperplane for separating intronless and intron-containing genes can be obtained by implementing SVM classifier based on this 7-dimensional feature space. Figure 1 and Figure 2 show the linearity of log-log plots of one intron-containing gene (Z31371) and one intronless gene (A10909) on the value 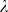, 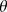, and 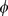. We can see that eigenvalues 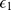, 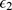, and 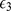 are perfectly fitted by the lines with slope 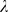, 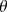 and 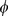 when 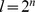, 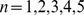.

**Table 1:** The seven feature parameters of 12 sample genes.

*Genbank Acce. No.*	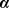	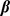	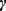	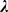	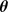	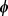	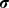
A00033	0.386	0.500	0.491	0.9933	0.9702	0.9415	0.2839
A17677	0.508	0.544	0.565	1.0757	1.0307	0.8789	0.2588
A11542	0.422	0.470	0.497	1.0431	1.0104	0.8742	0.2876
A22239	0.465	0.495	0.480	1.0673	1.0353	0.9351	0.2951
A24782	0.513	0.572	0.634	1.1773	1.1755	1.1214	0.3233
Z31371	0.500	0.526	0.558	1.0233	1.0336	1.0200	0.2732
M28289	0.576	0.635	0.632	1.3088	1.2991	1.0684	0.3462
V01510	0.575	0.617	0.716	1.3347	1.2171	1.0992	0.3429
U25810	0.560	0.657	0.623	1.2284	1.2740	1.1454	0.3340
M13580	0.480	0.622	0.624	1.2339	1.2114	1.0537	0.3337
U06674	0.507	0.498	0.555	1.1821	1.1609	0.9518	0.3274
J02989	0.518	0.577	0.537	1.2192	1.2463	1.0363	0.3495

### SVM parameter optimization

The kernel function 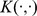 dominates the learning capability of the SVM [23]. We use radial basis kernel function 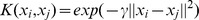 to predict the intronless from intron-containing genes. As in many multivariate applications, the performance of the SVM for classification depends on the combination of several parameters. In general, the SVM involves two classes of parameters: the penalty parameter 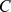 and kernel type 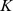. 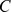 is a regularization parameter that controls the tradeoff between maximizing the margin and minimizing the training error. The kernel type 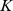 is another important parameter. In the radial basis function used in this study, 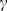 is an important parameter to dominate the generalization ability of SVM by regulating the amplitude of the kernel function. Accordingly, two parameters 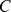 and 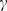 should be optimized. The parameter optimization is performed by using a grid search approach within a limited range. Prediction accuracy associated with mean-square-error (MSE) is used to select the parameters:
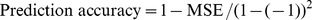



In the SVM classification, each data point represents a pair (geneID, 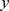); if the gene is experimentally intronless, 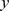 is assigned to 1, otherwise, 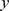 is 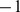.

### K-fold cross-validation

After all the seven parameters are determined, we can perform the *K*-fold cross-validation to estimate the accuracy of our predictive model. In a *K*-fold cross-validation, the original sample is randomly partitioned into 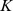 subsamples. Of the 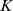 subsamples, a single subsample is retained as the validation data for testing the model, and the remaining 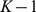 subsamples are used as training data. The cross-validation process is then repeated 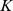 times (the folds), with each of the 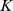 subsamples used exactly once as the validation data. Then the 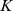 results from the folds are averaged (or otherwise combined) to produce a single estimation. Five-fold cross-validation is performed on this work. Using a grid search method, the model with best 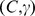 is obtained, which yields a minimum misclassification rate. The program implementing SVM comes from the R package “e1071” which is based on the libsvm 2.8 package [24]. The discriminant accuracy is defined as follows:
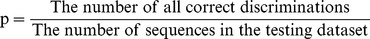



GENESCAN, N-SCAN, Z-curve method, and our DFA7 method are implemented on the dataset in order to compare the results. Since the output of these gene parsing and finding systems provides us with the (predicted) beginning and ending coordinates of exons/introns in these sequences, it is easy for us to determine whether or not the gene is intron-bearing based on the prediction. For the intronless genes, the prediction is regarded as “false” if it predicts the splice sites between exons and introns. This confirms the existence of introns. For the intron-containing genes, the prediction is regarded as “false” if prediction shows “single exon”. For the intron-containing or intronless genes which do contain exons, the prediction is regarded as “false” if the predicted answer is “no exon”. By this way, the programs of GENESCAN and N-SCAN are performed directly on the testing set.

## Results and Discussion

### On 2000 mixed prokaryotic and eukaryotic genes

We test our DFA7 method on a large dataset which contains 1000 intronless genes (from prokaryotic genomes completely) selected randomly from UniProtKB/Swiss-Prot (release 15.1) and 1000 intron-containing genes selected randomly from Genbank database (release 170). These genes come from human, thale cress, mus musculus, and other eukaryotes in order to avoid similarity. The classical gene parsing systems GENSCAN, N-SCAN, Z-curve method, and DFA7 method are implemented. To avoid the bias of the discriminant accuracy defined in [7] and the similarity of testing dataset, five-fold cross-validation is used. In SVM classification, the parameter ranges are given as follows: 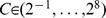, 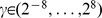. The prediction error profile has a minimum value at 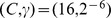, indicating that the optimal values of 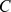 and 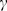 to construct the SVM model are 16 and 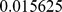, respectively.

**Figure 3:**
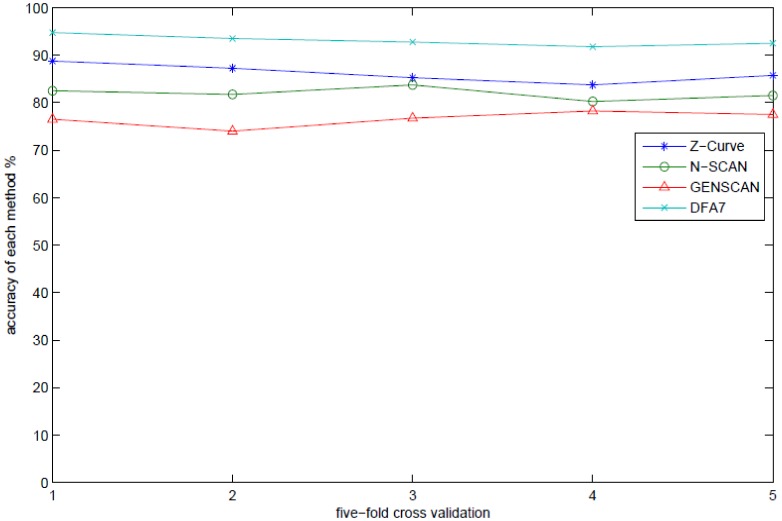
The accuracy comparison of DFA7 and other three methods on 2000 mixed prokaryotic and eukaryotic genes.

Using the optimal values of 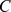 and 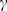, the prediction model is constructed based on the training set by using the SVM learning algorithm with RBF. To minimize data dependence on the prediction model, five-fold cross-validation sampling method is prepared. Each training set consists of 1600 sequences; half of them are randomly selected from data of intronless sequences, and the other half are randomly selected from data of intron-containing sequences. Each testing set is constructed using the left 400 sequences. The prediction results are listed in Table 2 and Figure 3. In Table 2, one can see that our DFA7 approach has higher accuracy than Z-Curve, N-SCAN, and GENSCAN methods.

**Table 2:** Prediction results of different methods on 2000 mixed prokaryotic and eukaryotic genes (%).

Methods	1 2 3 4 5	average
GENSCAN	76.50 74.00 76.75 78.25 77.50	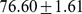
N-SCAN	82.50 81.75 83.75 80.25 81.50	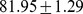
Z-Curve	88.75 87.25 85.25 83.75 85.75	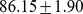
DFA7	94.75 93.50 92.75 91.75 92.50	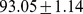

### On 1000 eukaryotic genes

In order to further illustrate the efficiency of our approach we test our method on another dataset. This carefully-selected dataset contains 1000 genes: 500 of them are human intronless genes which are randomly chosen from Intronless Gene Database [25], and the other 500 are intron-containing genes which are randomly chosen from Genbank database (release 170). Here we must emphasize that all the 1000 genes are from eukaryotic organisms. We partition the 1000 sequences into 5 parts; each part contains 200 sequences (100 intronless genes and 100 intron-containing genes). We treat each part as testing dataset, and the corresponding leftover (800 sequences) as training dataset.

We test two models: (1) using all 7 parameters (our DFA7 method), (2) using the first 3 parameters only (Z-Curve method). For each model, we firstly run 10-fold cross-validation on training dataset to get the best SVM tuning parameters 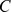 and 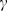, then fit the SVM model with the chosen parameters 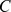 and 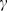, and finally use the fitted SVM model to predict the labels of training dataset (get training errors) and the labels of testing dataset (get testing errors). We show the test results in Table 3. We can see that, for the gene dataset from eukaryotic organisms, our DFA7 method still has higher accuracy than Zhang et al. 's method.

**Table 3:** Prediction results of different methods on 1000 eukaryotic genes.

	DFA7 Method	Z-Curve Method
Average error counts on 800 training genes	148.4	217.0
Average error counts on 200 testing genes	50.4	58.8
Average error counts on total dataset	198.8	275.8
Average accuracy rate	(1000−198.8)/1000 = 80.12%	(1000−275.8)/1000 = 72.42%

We also compare our DFA7 method with GENSCAN, which can predict the locations and exon-intron structures of genes in genomic sequences from a variety of organisms. We use the same dataset partitions as before (the 1000 sequences into 5 parts; each part contains 200 sequences: 100 intronless genes and 100 intron-containing genes) for this program. In Table 4, we can see that, GENSCAN seems to have higher accuracy (82.50%) than ours (80.12%). However, when we are using GENSCAN, some parameters (for example, organism) are needed to be specified. There are only three organisms to choose from: Vertebrate, Arabidopsis, and Maize. Since our datasets are from eukaryotes (actually, most sequences are from human), we choose organism parameter as “Vertebrate”. In this case, in order to get high-accuracy result for GENSCAN, we must know the prior information for the sequences, at least the hosts of these genes. Otherwise, for example, if we choose “Maize” as the organism parameter, GENSCAN got much lower accuracy (62.60%) as shown in Table 4. Thus it is a disadvantage for GENSCAN method. On the contrary, our DFA7 method does not need any prior information for the gene sequences. The 7 parameters are 7 natural quantities from the original DNA sequence, not set by any artificial intervention.

**Table 4:** Prediction results of GENSCAN on 1000 eukaryotic genes.

	GENSCAN-Vertebrate	GENSCAN-Maize
Partition 1	31	80
Partition 2	38	72
Partition 3	31	78
Partition 4	33	57
Partition 5	42	87
Total error counts	175	374
Average accuracy rate	(1000−175)/1000 = 82.50%	(1000−374)/1000 = 62.60%

### On 1200 eukaryotic genes

We also test our approach on another large dataset including 600 intronless genes and 600 intron-containing genes from three very different eukaryotic genomes (human, drosophila, and yeast). The 600 intronless genes include 200 human genes, 200 drosophila genes, and 200 yeast genes. Similarly, the 600 intron-containing genes also include 200 human genes, 200 drosophila genes, and 200 yeast genes. These genes are chosen from Intronless Gene Database [25], Berkeley Drosophila Genome Project [26], and Saccharomyces Genome Database [27]. We partition the 1200 sequences into 5 parts; each part contains 240 sequences (120 intronless genes and 120 intron-containing genes). We treat each part as testing dataset, and the corresponding leftover (960 sequences) as training dataset.

We test two models: (1) using all 7 parameters (our DFA7 method), (2) using the first 3 parameters only (Z-Curve method). For each model, we firstly run 10-fold cross-validation on training dataset to get the best SVM tuning parameters 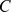 and 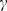, then fit SVM model with the chosen parameters 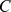 and 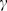, and finally use the fitted SVM model to predict the labels of training dataset (get training errors) and the labels of testing dataset (get testing errors). We show the test results in Table 5. We can see that, for the discriminant accuracy, our DFA7 method still largely outperforms Zhang et al. 's method.

**Table 5:** Prediction results of different methods on 1200 eukaryotic genes.

	DFA7 Method	Z-Curve Method
Average error counts on 960 training genes	204	266.2
Average error counts on 240 testing genes	62.6	74.4
Average error counts on total dataset	266.6	340.6
Average accuracy rate	(1200−266.6)/1200 = 77.78%	(1200−340.6)/1200 = 71.62%

Furthermore, we compare our DFA7 method with GENSCAN with the same dataset. When we are using GENSCAN, the parameter organism is needed to set. If we choose the organism parameter as “Vertebrate”, the average accuracy rate for this dataset is only 40%. Actually, the genes in this dataset are from three very different eukaryotic organisms: mammalian (human), invertebrate (drosophila), and unicellular (yeast). The diversity of our dataset leads to the very low accuracy rate. Therefore, the GENSCAN can not output meaningful prediction results with the genes from very diverse hosts. However, our approach can be used on a universal dataset.

### Examine DFA7 method by one-by-one feature deletion

To test whether there is any overfitting issue, we use the one-by-one feature deletion to justify our DFA7 method. Here we use the previous dataset of 1000 eukaryotic genes. We randomly divide the 500 intronless sequences into 250 and 250, and randomly divide 500 intron-containing sequences into 250 and 250, then use 250 intronless and 250 intron-containing genes as training dataset, and use the rest 250 intronless and 250 intron-containing genes as testing dataset. Thus, in this case, the training dataset and the testing dataset are totally independent.

For one-by-one feature deletion, we test 8 models: (1) using all 7 parameters, (2) using 6 parameters after deleting 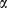, (3) using 6 parameters after deleting 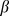, (4) using 6 parameters after deleting 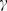, (5) using 6 parameters after deleting 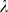, (6) using 6 parameters after deleting 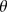, (7) using 6 parameters after deleting 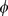, (8) using 6 parameters after deleting 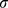. For each model, we run 10-fold cross-validation on training dataset to get the best SVM tuning parameters C and “
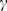
”, fit SVM model with the chosen parameters C and “
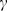
”, then use the fitted SVM model to predict the label of training dataset (get training errors) and the label of testing dataset (get testing errors). We show the test results in Table 6.

**Table 6:** Prediction results of 1000 eukaryotic genes based on DFA7 method by one-by-one feature deletion testing.

	All 7 parameters	deleting 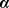	deleting 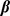	deleting 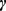	deleting 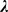	deleting 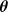	deleting 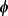	deleting 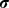
Average error counts on 500 training genes	72.2	89.4	99.4	87.6	76.0	68.2	88.6	73.4
Average error counts on 500 testing genes	130.0	131.4	126.0	141.2	129.4	132.8	128.4	129.2
Average error counts on total dataset	202.2	220.8	225.4	228.8	205.4	201.0	217.0	202.6
Average accuracy rate	79.78%	77.92%	77.46%	77.12%	79.46%	79.90%	78.30%	79.74%

From the results, we can see that, except “deleting 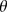
”, the model using all 7 parameters gives the highest average accuracy in Table 6. Here we must point out that 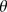 is not an overfitting parameter. The model of “deleting 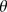
” gives more error counts than the original DFA7 model for many partitions. However, there is only one random partition dataset in which the “deleting 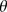
” model gives much less errors than DFA7. This big difference causes that the final average accuracy of the “deleting 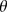
” model is slightly higher than the DFA7 model. Actually, for most random partitions of dataset, our DFA7 model gives much higher accuracies.

### Comparison with other machine learning approaches

Based on our proposed new features, we also use other machine learning techniques to test the classification results. Using the same dataset of 1000 eukaryotic genes, we compare the performance of SVM, Backpropagation network (BPN), and Radial Basis Function network (RBFN) [28]–[30]. To train a BPN, we use the function “neuralnet” in the R package “neuralnet”. To train an RBFN, we use the function “rbf” in the R package “RSNNS”. Both of them are available at http://cran.r-project.org/web/packages/.

The training errors and testing errors following the same setup of partitions as in section “On 1000 eukaryotic genes” are shown in Table 7 and Table 8, respectively. Here SVM7 indicates SVM with all the 7 parameters, SVM3 indicates SVM with the first 3 parameters, and so on. Note that BPN7 is not available because the convergence of training procedure of BP network with all the 7 parameters is too slow. Based on the cross-validation results, we can see that BPN and RBFN have much more errors than SVM on the dataset.

**Table 7:** Error counts for 800 eukaryotic genes with 5 partitions on 3 different machine learning methods.

	SVM7	SVM3	BPN3	RBFN3	RBFN7
Partition 1	170	223	298	296	246
Partition 2	178	211	319	287	279
Partition 3	129	213	306	286	235
Partition 4	151	216	315	274	232
Partition 5	114	222	306	284	237
Average error counts	148.4	217.0	308.8	285.4	245.8

**Table 8:** Error counts for 200 eukaryotic genes with 5 partitions on 3 different machine learning methods.

	SVM7	SVM3	BPN3	RBFN3	RBFN7
Partition 1	53	54	80	66	60
Partition 2	46	61	75	69	56
Partition 3	50	62	70	86	60
Partition 4	44	59	78	73	62
Partition 5	59	58	73	71	65
Average error counts	50.4	58.8	75.2	73.0	60.6

## Conclusion

In this work, we propose a new computational approach to distinguish between intron-containing and intronless gene sequences. In comparison with previous literature, the predictive performance of our method has been significantly enhanced. It is anticipated that the current method can be a complementary tool for distinguishing intronless genes from intron-containing genes. Seven feature parameters 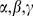, 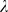, 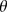, 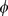, and 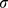 can be computed using the algorithmic steps as we described. SVM classifier with RBF function is also performed on those seven parameters to classify the genes. Our new feature parameters can be used to discover more information hidden within the genes. Our DFA7 method mainly focuses on distinguishing intron-containing and intronless gene sequences. Further studies of this method will be needed to make specific splice-site predictions available. We will also evaluate the relative importance of the feature parameters and find more valuable features, which could help to classify genes and proteins with higher accuracy based on their structures. The datasets used in this work are available at http://www.math.uic.edu/~jyang06/publications/datasets/.
